# Beyond the primer: anatomical site and cyst viability drive the molecular detection of *Taenia saginata* in slaughterhouse samples

**DOI:** 10.1186/s12917-026-05722-2

**Published:** 2026-07-28

**Authors:** Emilly Liandra Camargo Silva, Lara Pael Coelho, Jéssica Cristine K. Moriya, Ana Paula Figueiredo, Ana Clara Leonardo de Araújo, Raquel Soares Juliano, Fernando de Almeida Borges, Marcelo Fontes Pereira, Marcio Roberto Silva, Paulo Henrique Duarte Cançado, Lenita Ramires dos Santos, Flábio Ribeiro de Araújo

**Affiliations:** 1 Postgraduate Program in Veterinary Sciences, Universidade Federal de Mato Grosso do Sul (UFMS), Campo Grande, MS 79070-900 Brazil; 2 Embrapa Beef Cattle/Ministry of Agriculture, Livestock and Food Supply Scholarship, Campo Grande, MS 79106-550 Brazil; 3 Conselho Nacional de Desenvolvimento Científico e Tecnológico (CNPq) Scholarship/Embrapa Beef Cattle, Campo Grande, MS 79106-550 Brazil; 4 Conselho Nacional de Desenvolvimento Científico e Tecnológico (CNPq)/PIBIC Program/Embrapa Beef Cattle, Campo Grande, MS 79106-550 Brazil; 5 Embrapa Pantanal, Corumbá, MS 79320-900 Brazil; 6 Private Veterinary Consultant, Campo Grande, MS 79000-000 Brazil; 7 Embrapa Dairy Cattle, Juiz de Fora, MG 36038-330 Brazil; 8 Embrapa Beef Cattle, Campo Grande, MS 79106-550 Brazil

**Keywords:** Bovine cysticercosis, *Taenia saginata*, PCR, Molecular diagnosis, Meat inspection, Food safety

## Abstract

**Background:**

Bovine cysticercosis, caused by the larval stage of *Taenia saginata*, results in significant economic losses for the beef industry due to carcass condemnation. While routine post-mortem inspection is mandatory, visual diagnosis lacks specificity, particularly for degenerated or calcified cysts. This study primarily aimed to evaluate how anatomical site and cyst viability influence the molecular confirmation of suspected lesions. As a secondary methodological objective, we assessed the performance of three conventional PCR assays—a widely used large subunit (LSU) rRNA target (328 bp), a previously described Cytochrome c oxidase subunit 1 (COX1) target (253 bp), and a novel COX1 target (428 bp) designed herein—to ensure primer selection did not bias our findings. A total of 232 suspect macroscopic lesions (180 calcified cysts, 12 viable cysts, and 40 non-specific lesions) were collected from cattle slaughtered in Mato Grosso do Sul, Brazil.

**Results:**

Our results demonstrated no statistically significant difference in diagnostic sensitivity among the three primer sets (*p* > 0.05), indicating that molecular confirmation is not limited by primer selection. Instead, molecular detection was profoundly driven by cyst viability and anatomical location (*p* < 0.001). While viable cysts yielded high positivity rates (up to 66.7%; 95% CI: 34.9%–90.1%), detection in calcified lesions dropped to approximately 28.9% due to severe DNA degradation. Crucially, calcified hepatic lesions exhibited drastically lower PCR positivity (11.4%–15.2%) compared to those in the heart (46.0%–54.0%) and head muscles (50.0%–60.0%).

**Conclusions:**

These findings indicate that the anatomical location of a lesion heavily dictates molecular detection rates. While visual inspection of hepatic tissue often conflicts with molecular results, negative PCR in the liver does not necessarily indicate the absence of infection. Rather, this discrepancy highlights a critical limitation in detecting highly degraded DNA, likely driven by rapid clearance within the enzymatically aggressive hepatic parenchyma, combined with the potential misclassification of non-parasitic granulomas. Due to the high rate of false negatives in calcified cysts, conventional PCR cannot be safely recommended for real-time carcass clearance. Instead, to improve diagnostic accuracy, epidemiological surveillance and retrospective sanitary audits should prioritize suspect lesions in the heart and striated muscles over those found in the liver.

## Background

Brazil plays a prominent role in the global beef market, consolidating its position as the world’s largest beef exporter [2]. In this context, strict hygienic–sanitary control throughout the production chain is essential to ensure food safety and maintain access to international markets. Among parasitic diseases affecting both public health and the meat industry, bovine cysticercosis—caused by the larval stage of the zoonotic cestode *Taenia saginata*—stands out as a major cause of partial or total carcass condemnation [14]. In Brazil, while recent large-scale surveillance encompassing nearly 22 million carcasses indicates a low overall national prevalence of 0.09%, official inspection records reveal sharp regional variations, with state-level prevalence reaching 0.51% in São Paulo and local hyper-endemic microregions exceeding 2.2%, triggering significant economic losses [6]. The taeniasis–cysticercosis complex represents a critical public health concern, directly involving the human–animal interface and environmental contamination through the shedding of infective eggs [7, 8].

In Brazil, according to the Regulation of Industrial and Sanitary Inspection of Products of Animal Origin (RIISPOA) [3], the diagnosis of bovine cysticercosis relies on post-mortem macroscopic identification, classifying cysticerci as viable or calcified. Although routine meat inspection is essential, visual examination presents inherent limitations. Degenerated or calcified cysticerci are notoriously difficult to distinguish from other pathological lesions. Consequently, misclassification frequently occurs, resulting in the overestimation of infection rates and the inappropriate condemnation of carcasses [10].

Recent studies reinforce these diagnostic challenges. In the state of Tocantins, Brazil, visual inspection identified a low frequency of cystic lesions, yet molecular analysis and histopathology confirmed *T. saginata* in only a fraction of these samples, identifying many as non-specific granulomatous lesions or hydatid disease [11]. Similarly, [10] demonstrated that visual inspection significantly overestimated the occurrence of *T. saginata* cysticerci in cattle slaughtered in Egypt when compared to PCR confirmation. Together, these findings highlight the limited specificity of macroscopic diagnosis and emphasize the need for complementary approaches.

Molecular techniques, particularly polymerase chain reaction (PCR), are widely utilized for their high analytical sensitivity and specificity [5, 12, 18]. Nevertheless, the diagnostic sensitivity of PCR on field samples is inherently constrained by biological variables, such as target DNA degradation in mineralized cysts, and depends heavily on primer specificity [1, 18].

We hypothesized that while conventional PCR improves the specificity of visual diagnosis, its field performance is significantly influenced by biological factors, such as the anatomical origin and viability of the lesion, rather than technical variations in primer design. Accordingly, the primary objective of this study was to evaluate how anatomical site and cyst viability drive the molecular detection of *T. saginata* in slaughterhouse samples. As a secondary methodological objective, we validated and compared a novel conventional PCR assay designed herein against two previously described targets to ensure that primer selection did not bias our biological findings and to assess the practical applicability of molecular tools in retrospective sanitary surveillance.

## Methods

### Study design and sample collection

This study was conducted between April 2023 and June 2024 in the state of Mato Grosso do Sul, Brazil, to evaluate and compare the diagnostic performance of three conventional PCR assays for detecting *Taenia saginata* DNA in bovine tissue. A total of 232 tissue samples were analyzed. The majority of the samples (*n* = 224) were collected from cattle slaughtered at an establishment operating under the Federal Inspection Service (SIF), while the remaining samples (*n* = 8) were sourced from a slaughterhouse under the State Inspection Service (SIE). All samples were collected at the Final Inspection Department from carcasses that had been diverted due to suspected cystic lesions identified during routine post-mortem examination. Tissue fragments were excised from standard anatomical inspection sites, including the heart, liver, diaphragm, masseter and pterygoid muscles, and esophagus. Each sample was individually cataloged (recording carcass number, batch number, anatomical location, and collection date), placed in a sterile plastic container, maintained under refrigeration, and transported to the Animal Health Laboratory at Embrapa Beef Cattle for molecular analysis. To ensure sufficient material for DNA extraction, cystic structures were collected along with a small margin of adjacent tissue.

### Macroscopic classification

Routine post-mortem examination performed by the Federal (SIF) and State (SIE) services strictly followed official Brazilian guidelines, which rely on visual inspection and systematic incisions of target organs (heart, masseter muscles, tongue, and liver). Viability was determined macroscopically based on pathognomonic features: cysts were classified as viable when presenting as a translucent, fluid-filled vesicle containing a visible, invaginated white scolex. Lesions were classified as calcified when they presented as opaque, dry, caseous, or mineralized nodules lacking fluid and clear parasitic structures, without routine microscopic compression during the fast-paced slaughter line. Based on slaughterhouse records and visual inspection, the 232 collected samples were divided into three distinct categories: calcified cysts (*n* = 180, which included all eight samples from the state-inspected slaughterhouse), viable cysts (*n* = 12), and visually negative or inconclusive lesions (*n* = 40). This last group served as specificity controls and comprised non-specific macroscopic findings, such as hepatic fatty nodules, necrotic or inflammatory liver nodules, uncharacterized calcified liver nodules, unspecified cardiac nodules, focal connective tissue in the liver and heart, and hepatic sarcocystosis lesions.

### DNA extraction and quality assessment

Genomic DNA was extracted from approximately 25 mg of homogenized tissue using the PureLink Genomic DNA Mini Kit (Invitrogen, USA), according to the manufacturer’s instructions. DNA concentration and purity were determined using a NanoDrop 2000c spectrophotometer (Thermo Scientific, USA). DNA integrity was verified by electrophoresis on a 0.8% agarose gel.

### PCR assays

Three conventional PCR assays for the detection of *T. saginata* were evaluated. The first assay, designed and standardized in this study, targeted a 428-base pair (bp) fragment of the mitochondrial cytochrome c oxidase subunit 1 (*COX1*) gene. Specific primers were designed using the Primer-BLAST tool (NCBI): forward (5′-GGATTGCCTCGTCGTGTTTG-3′) and reverse (5′-CTAAGCATGATGCAAAAGGC-3′). Amplification reactions (20 µL) contained GoTaq Colorless Master Mix 2X (Promega, USA), 0.25 µM of each primer, and 200 ng of template DNA. Thermal cycling was performed in an MJ Mini Personal Thermal Cycler (Bio-Rad, USA), consisting of an initial denaturation at 95 °C for 10 min, followed by 35 cycles of 94 °C for 1 min, 51 °C for 1 min, and 72 °C for 40 s, with a final extension step at 72 °C for 7 min.

The second assay, previously described by [13], targeted a 328-bp fragment of the large subunit (LSU) rRNA gene using the primers TBR-3 (generic forward, 5’-GGCTTGTTTGAATGGTTTGACG-3’) and TBR-4 (*T. saginata*-specific reverse, 5’-CGACTCATGAAGATAAACAAGGT-3’). The PCR reactions (20 µL) were performed using the same reagent concentrations as the first assay. The cycling program consisted of an initial denaturation at 94 °C for 2 min, followed by 40 cycles of 94 °C for 30 s, 59 °C for 30 s, and 72 °C for 1 min, concluding with a final extension at 72 °C for 5 min.

The third assay, adapted from [9], amplified a 253-bp fragment of the *COX1* gene using the forward (5′-GGGTGCTGGTATAGGGTGGACT-3′) and reverse (5′-ACGTAAATAAATAAGCCCACAATATT-3′) primers. Amplification reactions (25 µL) contained GoTaq Colorless Master Mix 2X, 0.3 µM of each primer, 200 ng of template DNA, and nuclease-free water to reach the final volume. The cycling conditions included an initial denaturation at 94 °C for 4 min, followed by 35 cycles of 94 °C for 30 s, 59 °C for 1 min, and 72 °C for 1 min, with a final extension step at 72 °C for 7 min.

To assess the presence of PCR inhibitors in samples that tested negative for *T. saginata*, an internal control PCR targeting the bovine *DGAT1* gene was performed. This primer pair amplified a 282 bp fragment of this constitutive gene, confirming DNA quality and amplification competence. PCR products from all assays were separated by electrophoresis on 1.5% agarose gels in 1 × TBE buffer at 100–120 V. A 1 kb Plus DNA ladder (Invitrogen, USA) was used as a molecular size marker. Gels were stained with a commercial nucleic acid dye and visualized under ultraviolet light.

### Analytical sensitivity assessment

The analytical sensitivity of the standardized assay was evaluated using a 1:2 serial dilution of DNA extracted from viable *T. saginata* cysticerci (initial concentration of 200 ng/µL). The detection limit was defined as the lowest concentration that produced a clear, reproducible band on the 1.5% agarose gel.

### Statistical analysis

To evaluate the detection capability across different molecular targets, the positivity frequencies of three PCR primer sets were analyzed: COX1 (428 bp; designed in this study), LSU rRNA (328 bp), and a second COX1 target (253 bp). To determine if PCR positivity was significantly associated with the macroscopic classification of the lesions (viable cysts, calcified cysts, or other lesions), the Chi-square test or Fisher's Exact test was employed, depending on the expected cell frequencies. Furthermore, because the same 232 samples were evaluated across all three molecular targets (paired data), Cochran's Q test was used to assess whether there was a statistically significant difference in overall diagnostic sensitivity among the three primers. All statistical analyses were performed using R software version 4.3.2 [16]. A significance level of p < 0.05 was adopted for all analyses.

## Results

**Fig. 1:**
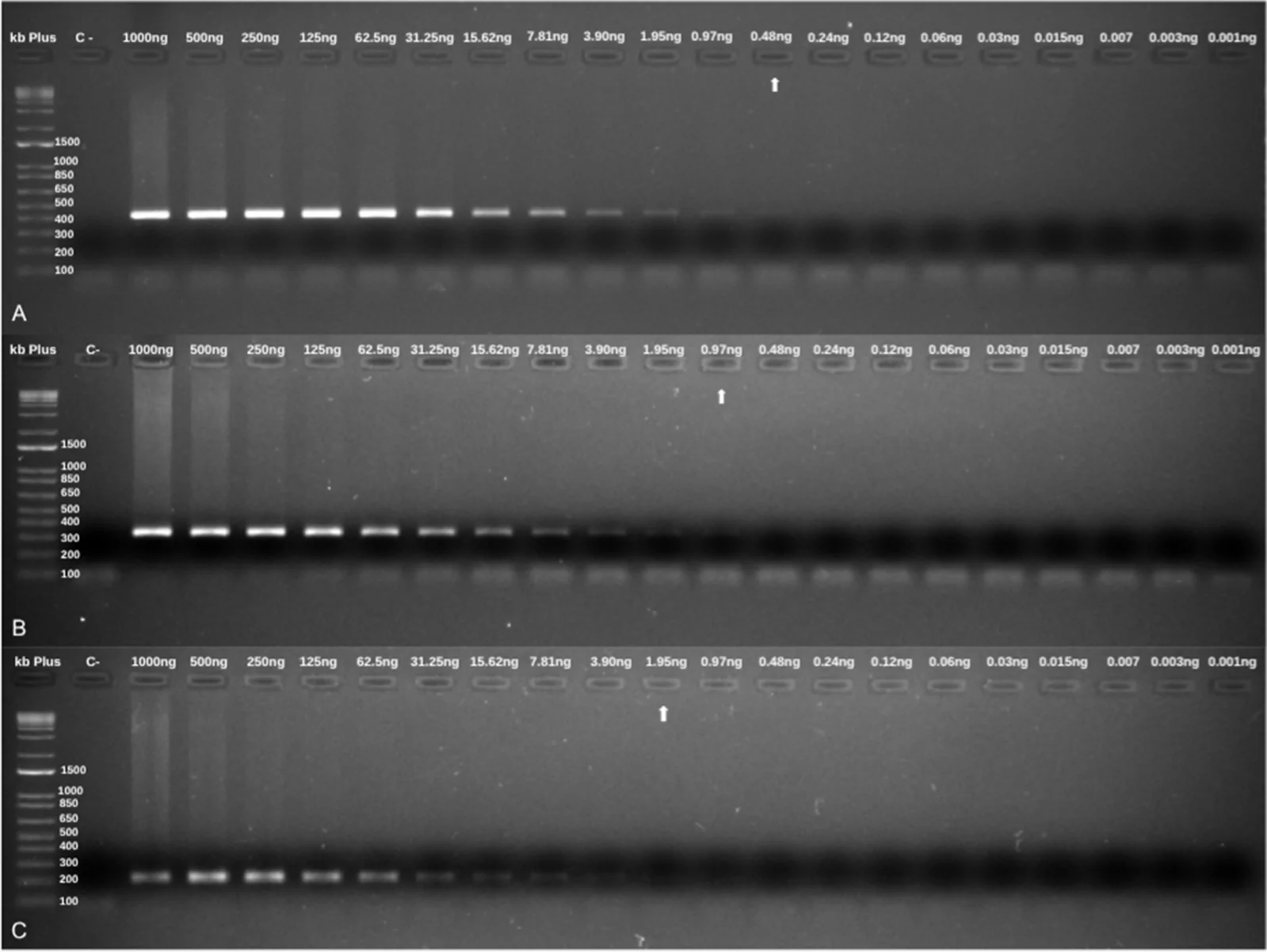
Placeholder: Comparative analytical sensitivity of primer sets for *Taenia saginata* detection

Evaluation of analytical sensitivity demonstrated the following detection limits: 0.48 ng for the COX1 (428 bp) primer pair, 0.97 ng for LSU rRNA (328 bp), and 1.95 ng for COX1 (253 bp). These results indicate that the novel COX1 (428 bp) assay yielded the highest analytical sensitivity (Fig. 1).

Molecular detection of *T. saginata* DNA varied significantly depending on the macroscopic developmental stage of the lesion, confirming the profound impact of cyst degeneration on PCR performance (*p* < 0.001). For viable cysts (*n* = 12), positivity rates were high, reaching 50.0% (95% CI: 21.1%–78.9%) for COX1 (428 bp), 58.3% (95% CI: 27.7%–84.8%) for LSU rRNA (328 bp), and 66.7% (95% CI: 34.9%–90.1%) for COX1 (253 bp). In contrast, detection in calcified cysts (*n* = 180) dropped substantially to 26.7%, 30.0%, and 28.9%, respectively, reflecting severe DNA degradation in chronic lesions. Samples macroscopically classified as 'other lesions' (*n* = 40) exhibited baseline positivity rates of only 2.5% to 5.0%, highlighting the high specificity of the assays.

When comparing the overall diagnostic performance of the three molecular targets across the 232 paired samples, the LSU rRNA target yielded the highest absolute number of positive detections (27.1%), followed by the 253 bp COX1 target (26.3%) and the 428 bp COX1 target (23.7%). However, Cochran's Q test revealed that these variations were not statistically significant (*p* > 0.05), indicating that no single molecular target demonstrated absolute diagnostic superiority over the others in routine field samples (Table 1).

**Table 1:** Comparison of PCR positivity across three molecular targets for the detection of *Taenia saginata* according to macroscopic lesion stage

Sample classification	Total (n)	COX1 (428 bp) [This study] Pos	COX1 (428 bp) [This study] Neg	LSU rRNA (328 bp) [Jardim et al.] Pos	LSU rRNA (328 bp) [Jardim et al.] Neg	COX1 (253 bp) [El-Dakhly et al.] Pos	COX1 (253 bp) [El-Dakhly et al.] Neg
Calcified cyst	180	48	132	54	126	52	128
Viable cyst	12	6	6	7	5	8	4
Other lesions*	40	1	39	2	38	1	39
**Total**	**232**	**55**	**177**	**63**	**169**	**61**	**171**

*Includes macroscopic findings such as various hepatic nodules (fatty, necrotic, inflammatory, calcified, and unspecified), cardiac nodules, connective tissue in the liver and heart, and hepatic sarcocystosis

*Abbreviations*: *LSU rRNA* Large Subunit Ribosomal RNA, *COX1* Cytochrome c oxidase subunit 1, *bp* base pairs, *Pos* Positive, *Neg* Negative

Crucially, organ-stratified analysis revealed that anatomical location profoundly impacts molecular confirmation rates, particularly for chronic lesions (*p* < 0.001). Among calcified cysts, samples from the liver (*n* = 105) exhibited drastically reduced PCR positivity (11.4% to 15.2%). In sharp contrast, calcified cysts in the heart (*n* = 50) and head muscles (*n* = 20) demonstrated substantially higher molecular positivity rates (46.0%–54.0% and 50.0%–60.0%, respectively). For viable cysts, found exclusively in the heart and masseter muscle, detection rates remained consistently high (66.7% to 77.8%). This marked discrepancy suggests that visual inspection of the liver suffers from low specificity (due to misclassification of non-parasitic granulomas) or that the hepatic microenvironment accelerates the degradation of parasite DNA (Table 2).

**Table 2:** Comparison of PCR positivity for *Taenia saginata* across three molecular targets according to organ and macroscopic lesion stage

Organ	Cyst Type	Total (n)	COX1 (428 bp) Pos	COX1 (428 bp) Neg	LSU rRNA (328 bp) Pos	LSU rRNA (328 bp) Neg	COX1 (253 bp) Pos	COX1 (253 bp) Neg
Liver	Calcified	105	16	89	13	92	12	93
Heart	Calcified	50	24	26	23	27	27	23
Heart	Viable	3	2	1	1	2	2	1
Masseter Muscle	Calcified	17	8	9	8	9	9	8
Masseter Muscle	Viable	9	6	3	5	4	5	4
Pterygoid Muscle	Calcified	3	2	1	2	1	3	0
Diaphragm	Calcified	1	1	0	0	1	1	0
Mixed Samples*	Calcified	4	1	3	2	2	2	2
**TOTAL**		**192**	**60**	**132**	**54**	**138**	**61**	**131**

*Mixed samples represent combined adjacent tissues occasionally collected from highly calcified or diffuse areas

*Abbreviations*: *LSU rRNA* Large Subunit Ribosomal RNA, *COX1* Cytochrome c oxidase subunit 1, *bp* base pairs, *Pos* Positive, *Neg* Negative

## Discussion

Routine post-mortem inspection of bovine carcasses remains the primary line of defense against the transmission of *T. saginata* [17]. However, the subjective nature of macroscopic evaluation often leads to diagnostic uncertainty, particularly with degenerated or calcified lesions [10]. The present study evaluated the diagnostic performance of three conventional PCR assays, revealing that while molecular tools are highly specific, their sensitivity in the field is profoundly dictated by the developmental stage of the cyst and its anatomical location.

Consistent with previous literature [4], our results demonstrated a significant decline in PCR positivity as cysticerci degenerate. Viable cysts contain intact parasitic cells with highly preserved DNA, making them ideal targets. In contrast, calcification represents the end-stage of a severe host-parasite inflammatory response [15]. Macroscopically and histologically, degenerated cysts are characterized by caseous necrosis, severe cellular infiltration, and eventual mineralization, culminating in the loss of identifiable parasitic features [8]. This caseous necrosis physically entraps remaining structures and severely degrades nucleic acids. Consequently, the lower molecular detection rate in calcified lesions represents a biological limitation of the sample (due to extensive DNA degradation or non-parasitic etiologies) rather than a technical failure of the PCR assays. In these cases, a negative PCR result represents a true negative for *Taenia saginata*, accurately reflecting the absence of amplifiable parasite DNA.

The high specificity of the assays was corroborated by the minimal positivity (2.5%–5.0%) observed in the 'other lesions' category [12]. Given the high analytical specificity of the PCR targets, we hypothesize that these few positive detections are not false positives, but rather represent true *T. saginata* infections presenting as atypical macroscopic lesions. During early parasitic migration or in cases of atypical localized host inflammatory responses, early lesions may manifest as non-specific focal connective tissue or uncharacterized nodules, leading to macroscopic misclassification on the slaughter line.

The novel COX1 (428 bp) primer set was designed to optimize analytical sensitivity (achieving a detection limit of 0.48 ng) and to offer an alternative mitochondrial target for epidemiological screening. By comparing its performance with previously established protocols and finding no statistically significant differences in field sample detection (*p* > 0.05), we ruled out primer-dependent bias. This cross-validation proves that the primary bottleneck in bovine cysticercosis molecular diagnosis is sample-related (DNA preservation) rather than technical, validating the newly designed COX1 (428 bp) primers as a reliable, interchangeable tool for future epidemiological studies.

The most striking and novel finding of this study emerged from the organ-stratified analysis. The profound discrepancy in molecular confirmation between calcified cysts in the liver (11.4%–15.2%) and those in the heart and head muscles (46.0%–60.0%) provides a critical biological explanation for the low agreement between visual inspection and PCR. This stark contrast supports two primary hypotheses. First, visual inspection of the liver suffers from inherently low specificity. As a major filtering organ, the liver is susceptible to various pathological insults resulting in granulomatous and calcified lesions (e.g., hepatic fascioliasis, hydatidosis, bacterial abscesses). During the rapid pace of an industrial slaughter line, these non-parasitic granulomas are easily misclassified as calcified *C. bovis* [4].

These observations align with previous reports analyzing the macroscopic profile of cysticercosis. [15] highlighted that hepatic cysticerci are predominantly non-viable (71.0% to 87.5%). Furthermore, [4] emphasized that non-viable cysticercus lesions are easily confused with other etiologies, reducing the specificity of gross anatomical examination. This explains why high visual occurrence rates of hepatic cysticerci often correspond to low true-positive rates upon histological or molecular confirmation [15].

Second, distinct tissue microenvironments likely dictate DNA preservation. The liver is an enzymatically aggressive environment with a robust local immune system (e.g., Kupffer cells). It is highly probable that the clearance of parasitic DNA occurs at a much faster rate in the hepatic parenchyma than in striated muscle tissue, where calcified granulomas may retain remnant DNA for extended periods.

These findings have direct economic implications for the global beef industry. Relying solely on macroscopic inspection for hepatic lesions leads to a massive overestimation of bovine cysticercosis, resulting in the unnecessary condemnation or costly cold-treatment of carcasses possessing only non-specific hepatic scars. By demonstrating that the vast majority of PCR-negative "calcified cysts" are clustered in the liver, this study advocates for a rigorous reevaluation of sanitary condemnation criteria.

Given that the overall molecular sensitivity for calcified cysts—the most frequent finding in slaughterhouses—was below 30%, conventional PCR cannot be safely recommended as a real-time complementary tool to clear carcasses during routine inspection. The risk of false negatives due to DNA degradation is too high for immediate food safety decisions. Instead, the true utility of this molecular protocol lies in epidemiological surveillance, retrospective sanitary audits, and forensic validation of specific abattoir batches.

Practical Implications in the Industrial Setting: The logistical constraints of high-speed slaughter lines are inherently incompatible with multi-hour molecular assays. Therefore, we do not propose altering the routine operational flow established by RIISPOA, which must continue to rely on visual macroscopic inspection for immediate carcass destination. Instead, this molecular approach serves as an off-line official monitoring tool. It can be utilized by the Federal Inspection Service (SIF) to periodically audit diagnostic accuracy, investigate specific high-prevalence batches, or validate the epidemiological status of specific supplier regions without impeding industrial line speeds.

Despite these robust findings, this study has certain limitations that warrant consideration. A critical limitation of this study is the absence of a histopathological gold standard. Without histological confirmation, we cannot definitively conclude whether the high rate of PCR-negative hepatic samples stems primarily from advanced DNA degradation in the liver parenchyma or from the macroscopic misclassification of non-parasitic granulomas. Therefore, our conclusions regarding the absence of the parasite in these specific samples must be interpreted as a robust hypothesis requiring future histological validation. The relatively low number of viable cysts (*n* = 12) compared to calcified lesions (*n* = 180) reflects the natural frequency of chronic infections encountered in slaughterhouse lines, but warrants caution; as reflected by the wide confidence intervals, the reported percentages are highly sensitive to minor fluctuations due to the limited statistical power of this specific cohort. Additionally, the initial sample screening relied on the routine visual inspection conducted by local abattoir staff. While this accurately reflects real-world industry practices and validates the need for molecular confirmation, it inherently carries the subjectivity of visual triaging. Future studies incorporating randomized carcass sampling regardless of gross macroscopic findings could further elucidate the true baseline prevalence of *T. saginata*.

## Conclusions

Conventional PCR is a highly specific tool for molecular confirmation, though its diagnostic sensitivity in field applications is intrinsically limited by the severe DNA degradation characteristic of calcified cysts. Our findings validate the newly designed COX1 (428 bp) primer set as a reliable alternative to previously established targets.

Crucially, this study reveals that the anatomical location of a lesion heavily dictates molecular detection rates. While the vast majority of diagnostic discrepancies between visual inspection and PCR originate in the liver, negative molecular results from calcified hepatic cysts do not necessarily indicate the absence of infection. Rather, this discrepancy highlights a critical limitation in detecting highly degraded DNA. It is highly likely driven by the rapid clearance of parasitic DNA within the enzymatically aggressive hepatic parenchyma, combined with the potential misclassification of non-parasitic granulomas. Therefore, low molecular detection rates in the liver must be interpreted with caution, as they reflect the biological limitations of the organ's microenvironment as much as the limits of macroscopic specificity.

Due to the high rate of false negatives in calcified cysts and the time required for laboratory processing, conventional PCR cannot be used for real-time carcass clearance on the slaughter line. Instead, this molecular tool has immediate practical application in retrospective sanitary audits, high-throughput epidemiological surveillance, and the validation of macroscopic inspection accuracy. While this molecular tool cannot be used to overrule individual carcass condemnation decisions due to the documented risk of false negatives in calcified hepatic lesions, its application at the macro-level allows for the auditing of visual inspection trends. By identifying regions or supplier farms with high frequencies of non-parasitic granulomas, it provides the epidemiological data necessary to refine systemic sanitary risk profiles and mitigate broader, unwarranted commercial impacts on cattle herds.

## Data Availability

The datasets generated and/or analyzed during the current study are available from the corresponding author on reasonable request.
